# Testicular epidermoid cysts: a reevaluation

**DOI:** 10.1186/s12894-019-0477-1

**Published:** 2019-06-11

**Authors:** Petra Anheuser, J. Kranz, E. Stolle, D. Höflmayer, F. Büscheck, S. Mühlstädt, G. Lock, K. P. Dieckmann

**Affiliations:** 1 Klinik für Urologie, Albertinen-Krankenhaus, Hamburg, Germany; 2 Urologische Klinik AK Wandsbek, Hamburg, Germany; 3 Klinik für Urologie und Kinderurologie, St.-Antonius Hospital, Eschweiler, Germany; 4 Institut für diagnostische und interventionelle Radiologie, Albertinen-Krankenhaus, Hamburg, Germany; 5 Institut für Pathologie, Universitätsklinikum Eppendorf, Universität Hamburg, Hamburg, Germany; 6 Universitätsklink und Poliklinik für Urologie, Martin-Luther-Universität, Halle-Wittenberg, Halle (Saale), Germany; 7 Klinik für Innere Medizin II, Albertinen-Krankenhaus, Hamburg, Germany; 8 Hodenzentrum Hamburg, Asklepios Klink Altona, Hamburg, Germany

**Keywords:** Testicular neoplasm, Germ cell tumour, Epidermoid cyst, Testis sparing surgery, Scrotal sonography

## Abstract

**Background:**

Testicular epidermoid cysts (TECs) are rare benign testicular neoplasms. As TECs are rarely associated with germ cell tumours (GCTs), the understanding of biological behaviour and clinical management of TEC is unresolved.

**Methods:**

We retrospectively searched the files of patients treated for testicular neoplasms and germ cell cancer in the time from 2000 to 2017. Those with TEC were subjected to closer review looking to clinical and histological features, and to results from imaging with ultrasonography (US), contrast enhanced sonography (CEUS) and magnetic resonance imaging (MRI).

**Results:**

Among 589 patients undergoing surgery for testicular tumour, nine simple TECs were identified (1.5, 95% confidence intervals 0.53–2.50%). Median age was 26 years. Imaging revealed sharply demarcated roundish lesions with avascular central areas. Eight patients underwent testis-sparing excision with no recurrence ensuing. One had orchiectomy because of large size of the mass. Histologically, TECs consisted of cornifying squamous cell epithelium and no accompanying germ cell neoplasia in situ. Two additional cases (0.3% of all) required orchiectomy because these TECs were associated with ipsilateral GCT.

**Conclusions:**

TEC is usually a benign lesion that can safely be diagnosed with US, CEUS and MRI due to its roundish shape and its avascular centre. Histologically, this TEC corresponds to the prepubertal-type teratoma unrelated to germ cell neoplasia in situ of the 2016 WHO classification. The other subtype of TEC that is associated with invasive GCT represents a teratoma of postpubertal-type. From a clinical point of view it could be easier to differentiate between a “simple TEC” which is benign (prepubertal type) and a “complex TEC” which is malignant because of its association with invasive GCT.

## Background

Testicular epidermoid cysts have been first reported in 1942 [1], but their histogenetic origin has been a matter of dispute ever since. Accordingly, clinical management has been a matter of controversy, likewise. In the recent WHO classification of 2016, testicular epidermoid cysts (TECs) are listed as teratoma of prepubertal type within the group of germ cell tumours unrelated to germ cell neoplasia in situ [2]. The ICD-O code 9084/0 denotes a benign behaviour of this neoplasm. However, TECs have also been documented as part of invasive testicular germ cell tumours (GCTs).Therefore it has been hypothesized that two different types of TECs exist, one a truly benign “simple” testicular EC and the other a “complex” TEC associated with GCT thus representing teratoma [4]. Due to the rarity of TECs there are only few systematic clinical evaluations available [4–6]. Most of the current knowledge is based on small clinical series and single case reports. To improve the over-all understanding of TECs we retrospectively reviewed our experience with simple TECs and with complex ECs.

## Methods

We retrospectively searched the files of all patients who underwent treatment for testicular neoplasms or germ cell cancer in the department of urology in Albertinen-Krankenhaus Hamburg in the time from January 2000 to March 2017. We looked into the histologies of the patients operated on testicular tumours and selected those with TEC for closer review. The relative incidence of TECs in relation to GCTs was determined by calculating proportions with 95% confidence intervals. Median age and side-localization of TECs were compared to findings in GCTs. Clinical and histopathological features as well as imaging findings of the TEC patients were tabulated and descriptively analysed. Ethical approval was given by the institutional ethical committee (Albertinen Ethikkommission: U3–2015).

## Results

**Fig. 1:**
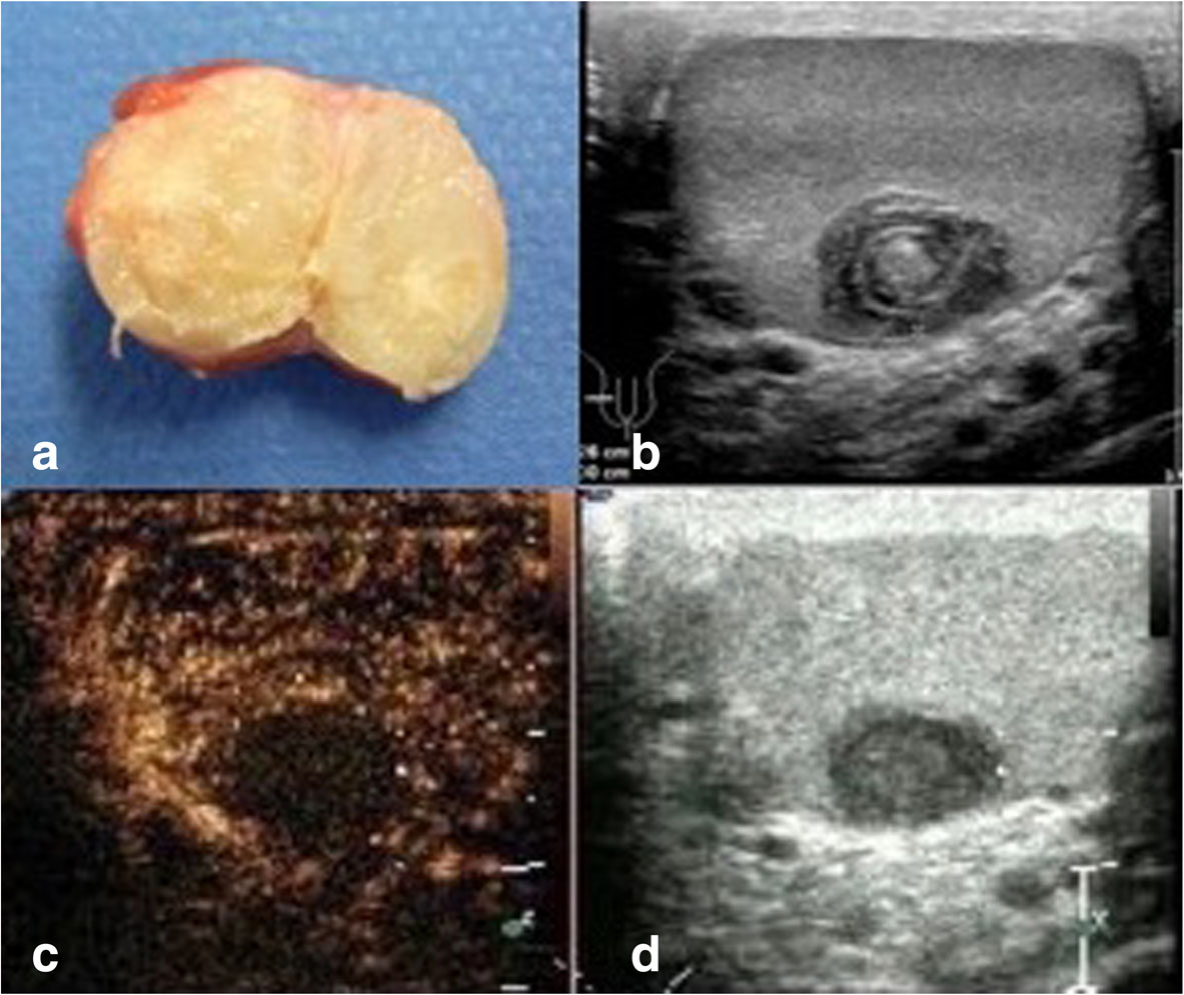
**a** Surgical specimen of excised simple testicular epidermoid cyst. Largest diameter of cyst 1.2 cm. Note the sharply demarcated rim of cyst and the yellowish amorphic mass inside. At the external side of the rim small layer of normal testicular tissue (brownish). **b** B-mode sonography of testis harbouring epidermoid cyst. Note the typical onion ring shape of the cyst core. **c**, **d** Contrast enhanced ultrasonography (CEUS) of testis with simple epidermoid cyst. Dual display with B-mode scan (right side of figure) and CEUS imaging (left side of figure). Note the absence of contrast material (air bubbles) in the centre of the cyst, indicating avascular area

A total of 625 patients were identified during the 17 year time-span, of whom 589 underwent surgery for a testicular tumour. The remainder were comprised of extragonadal GCTs as well as relapsing patients or other patients receiving upfront chemotherapy. Among the surgical cases, nine patients were identified with simple TEC (1.5, 95% CI 0.53–2.50%) We also identified 2 patients (0.3, 95% CI 0–0.81%) with testicular epidermoid cysts in association with ipsilateral germ cell tumour (“complex TECs”). Median age of the patients with simple TEC was 26 years (range 11–34 years). Noteworthy: 2 patients were of prepubertal age and both were submitted to our department because of its status as testicular cancer unit. Otherwise the department does not offer pediatric services. Median age of the GCT patients was 36 years (interquartile range 31–45 years) which is not significantly different from the median age of TEC patients. The right side was afflicted in six patients (66.7, 95% CI 29.93–92.51%). In GCTs there was also a slight predominance of the right side (50.51, 95%CI 45.56–55.46%). Localization is not significantly different between the two groups because the confidence intervals are widely overlapping. The presenting symptom of the TECs was a palpable painless nodule in 8 cases. Remarkably, two patients reported extraordinarily long symptomatic intervals of 10 and 13 years, respectively. One TEC case was an incidental finding upon surgery for suspected spermatic cord torsion. The mean size of the lesions was 17.4 mm (ranging from 6 to 40 mm). Tests sparing surgery (TSS) was performed in 8 patients (Fig. 1a), only in the case with the 40 mm mass was an orchiectomy required. AFP and βHCG were negative in all cases. In three of the more recent cases, microRNA-371a-3p was measured in serum. Likewise, there was no elevation of this novel marker.

**Fig. 2:**
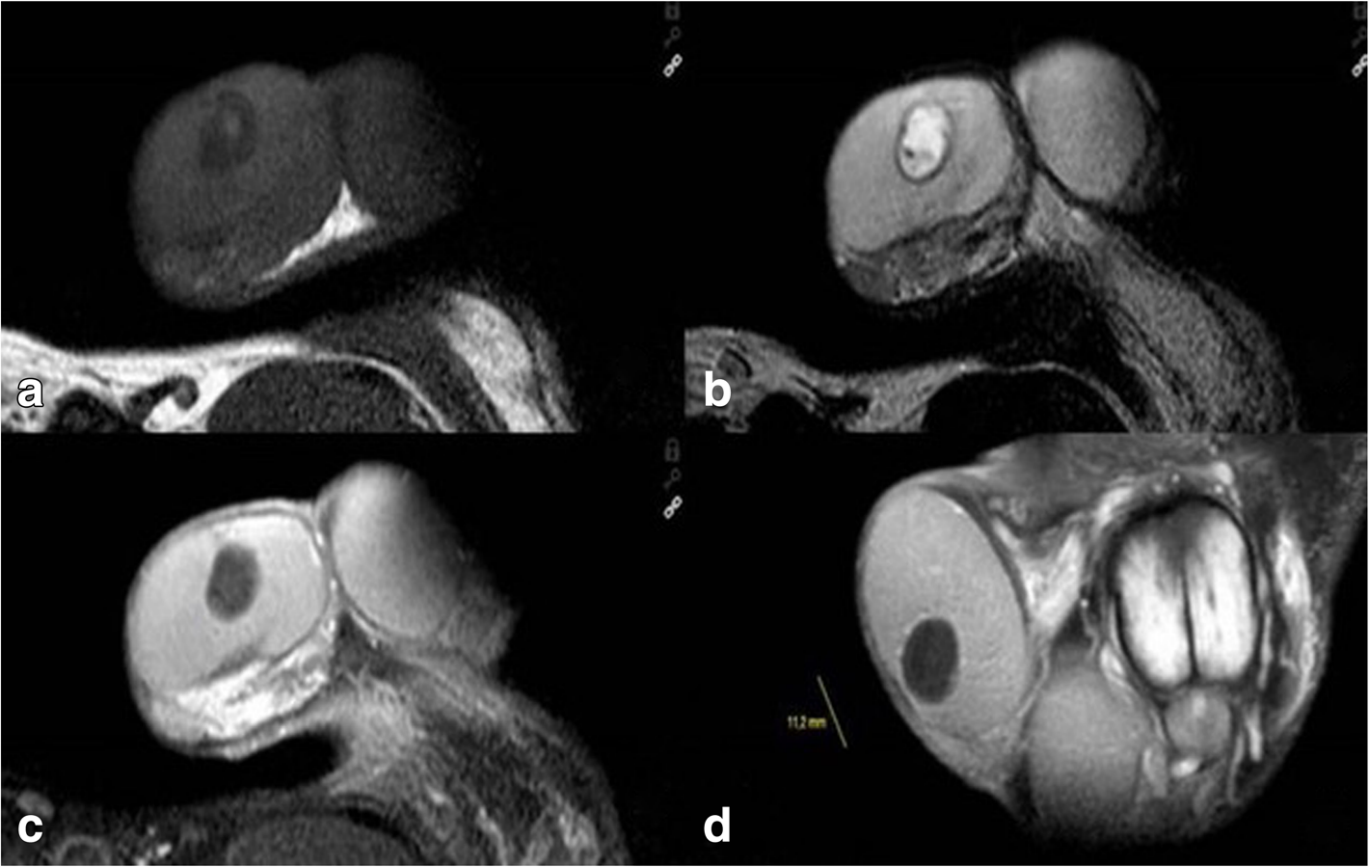
Scrotal MRI of patient with simple testicular epidermoid cyst, 1.5 Tesla MRI, surface coil. **a** T1 weighted imaging showing cyst with lower signal intensity than testicular parenchyma. **b**. T2-weighted imaging: typical bull-eye appearance of epidermoid cyst. Note the high signal intensity within cyst core. **c** and **d**. T1-weighted imaging with application of gadolinium-based contrast material. Note signal enhancement in testicular parenchyma but not in the cyst core highlighting the avascular area within the cyst

Regarding diagnostic procedures, all patients had scrotal ultrasound examination, 4 including contrast enhanced ultrasound (CEUS). Findings uniformly consisted of well demarcated roundish-shaped intratesticular lesions with a hyperechoic rim, no or little intralesional echogenicity and an acoustic shadowing on the posterior of the tumour. 5 patients had ring-shaped intralesional echogenicity corresponding to the onion-ring appearance (Fig. 1b). CEUS (all performed with 2.4 ml Sonovue, Bracco) demonstrated absence of contrast bubbles within the lesion indicating an avascular area (Fig. 1c, d). Scrotal magnetic resonance imaging (MRI) was available in 7 patients. Sharply demarcated bull-eyed intratesticular lesions were revealed in all cases with increased signal intensity at the rim of the lesions and no enhancement of the central area. T2-weighted imaging showed high intralesional signal intensity (Fig. 2a-d).

**Fig. 3:**
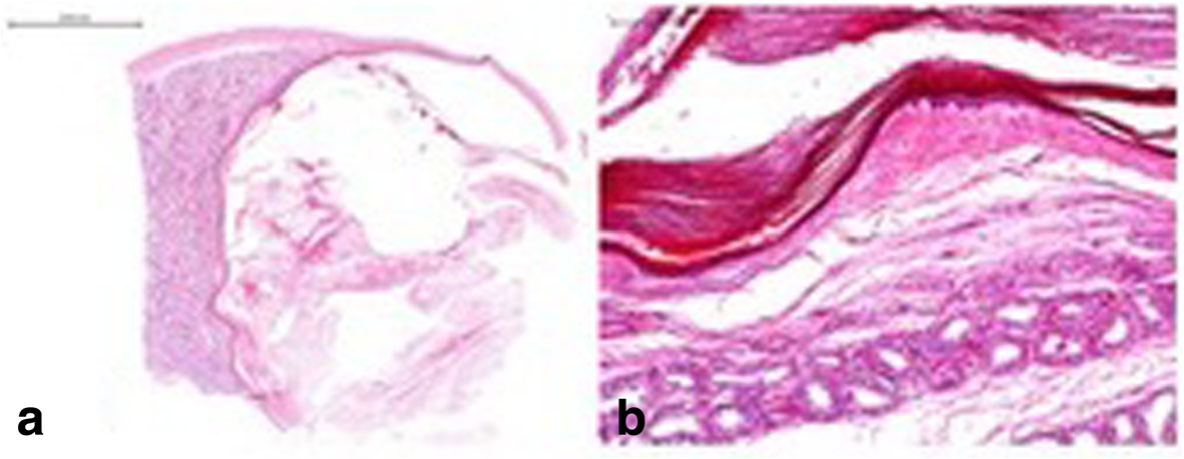
**a** Histological section of surgical specimen with excised testicular epidermoid cyst. Note: Cyst lumen (right side of figure) with layers of cornifying squamous epithelium and cell debris. On the right side normal testicular parenchyma. The cyst is surrounded by a capsule of fibrous tissue. Magnification scale at the upper left side. Hematoxylin eosin stain. **b** Testicular parenchyma with seminiferous tubules. No germ cell neoplasia in situ. of surgical specimen with excised testicular epidermoid cyst, same patient, higher magnification, see scale at the upper left side. Note: Cyst lumen (upper side of picture) with layers of cornifying squamous epithelium and cell debris. Testicular parenchyma with seminiferous tubules. No germ cell neoplasia in situ. Some tubules with spermatogenesis. Haematoxylin eosin stain

Histopathologically, all cases were classified as simple testicular epidermoid cysts with a surrounding fibrous membrane and layers of cornifying squamous epithelium as well as cell debris in the centre (Fig. 3a, b). No skin appendages and no GCNis were detected in any of these cases.

Follow-up is available in 7 patients with simple TEC. All of whom are well with no local recurrence after an average time-intervall of 6 years after surgery. One patient succumbed to cardiac failure, one was lost to follow-up.

**Fig. 4:**
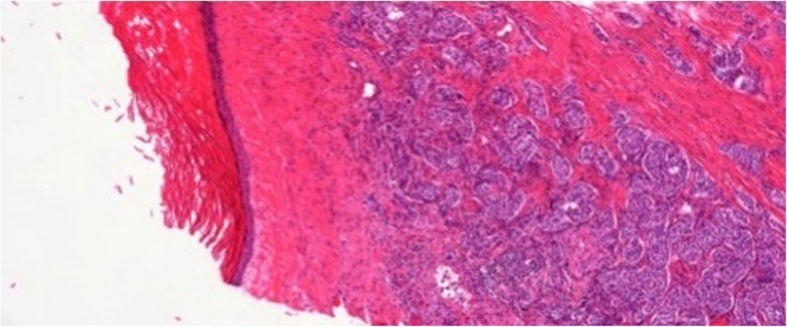
Histological section of complex testicular epidermoid cyst (left side of figure) in close vicinity to well-differentiated neuroendocrine tumor in testicular tissue outside of cyst (right side of figure). 5x magnification, hematoxylin-eosin stain

**Fig. 5:**
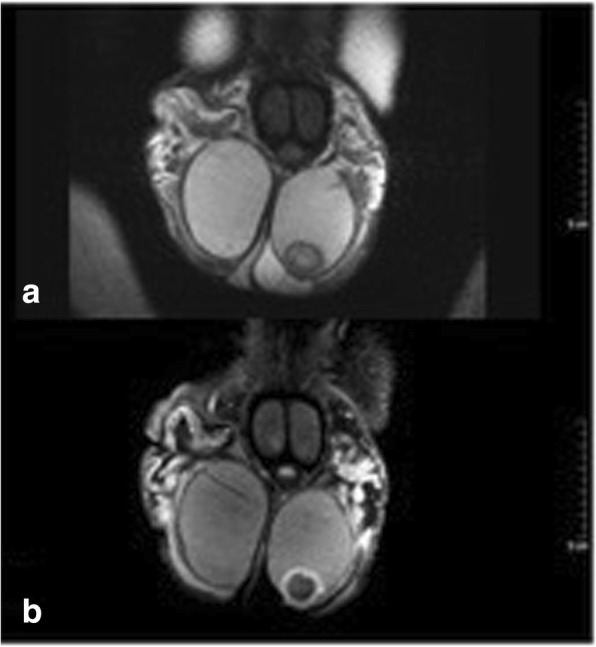
**a** Patient (adult) with complex Testicular epidermoid cyst, scrotal MRI, 1.5 Tesla, surface coil, coronar section, T1 weighted imaging without contrast material: sharply demarcated roundish cystic lesion at the caudal pole of the left testis. **b** Patient (adult) with complex Testicular epidermoid cyst, scrotal MRI, 1.5 Tesla, surface coil, coronar section, Gadolinium contrast material: roundish intratesticular cyst at the caudal testicular pole. Note the signal enhancement in the rim region of the cyst, no uptake of contrast material in the cyst centre

Two patients had complex epidermoid cysts, one of whom was associated with embryonal carcinoma. The second patient had synchronous bilateral tumours with teratoma (80%) embryonal carcinoma (15%) and yolk sac tumour (5%) on the right side and a neuroendocrine tumour, GCNis and an epidermoid cyst on the left (Fig. 4). In both cases the preoperative imaging detected only a simple TEC (Fig. 5a, b). Consequently, both underwent TSS initially and even intraoperatively, the lesions were considered simple cysts. Only after histopathological detection of typical GCT in the surrounding tissue, orchiectomy was performed in a second session. The classic markers were elevated in the second patient (18 years) but this elevation was clearly related to the contralateral mixed nonseminomatous GCT. Both patients received cisplatin-based chemotherapy in addition to surgery and remained disease-free thereafter.

## Discussion

Simple TEC is a rare but well-known lesion. As shown herein, TECs account for only 1.5% of all testicular neoplasms among post-pubertal men. This figure corresponds well with the 2.1% frequency reported previously [4, 5, 7] but it is clearly lower than the 10% relative incidence reported in a series from Taiwan [8]. Ethnic predisposition may account for that striking difference.

TECs have many clinical characteristics in common with GCT: Particularly clinical presentation with painless mass [9], the age predisposition of early adulthood and the preponderance of the right side. All these features were confirmed in our series. Reportedly, patients with TEC may have exceptionally long symptomatic intervals [10, 11]. Accordingly, two of our patients had noted a testicular nodule for as long as 10 and 13 years before surgery, respectively.

Usually, the diagnosis can be safely made with B-mode scrotal ultrasound. The typical onion-ring phenomenon is present in about 60% of all cases [12, 13]. The cyst-like appearance and the roundish configuration are further typical findings of the present and previous series [8, 14, 15]. A more distinctive finding is the absence of vascularization of the cyst core documented with CCDS (colour-coded duplex sonography) and CEUS as shown in four of our cases and previously reported by others [16]. Scrotal MRI can likewise detect these morphologic features [17, 18]. The avascular nature of the cyst core is safely shown by the absence of signal enhancement after application of gadolinium-based contrast material [19–21]. This finding was confirmed in all 7 patients of our series undergoing MRI.

Local excision (TSS) is the treatment of choice for TEC as realized in 8 of our patients. Orchiectomy is exceptionally required in cases with a large mass and only little remaining testicular tissue. Accordingly, one of our cases underwent orchiectomy. Similar cases are reported [6, 11]. Recurrences after TSS have not yet been experienced until now and the same is true for 7 of our patients in whom follow-up is available.

Histopathological findings in our series fully accord with previous reports. The lesion typically consists of a well-defined cyst lined by a fibrous membrane and filled with layers of cornifying squamous epithelium and cell debris. No skin appendages are found in the cyst’s lumen and no GCNis is present in the adjacent testicular parenchyma [22, 23].

The most striking finding of the present series is that simple TEC can obviously be mimicked by epidermoid cysts developing in association with full-blown GCT [24]. The association of TEC with ipsilateral GCT has been reported sporadically to date [3, 7, 25, 26]. The association of GCT with contralateral TEC has also been noted [27]. Urologic surgeons should be aware of the risk of concomitant GCT when conducting TSS of TEC. Therefore tumour marker measurements should be done preoperatively as is recommended for the examination of any scrotal mass [28]. As previously documented, the novel marker miR-371a-3p can substantially aid in diagnosing equivocal testicular masses [23]. Accordingly, miR371a-3p was elevated in both cases with complex TECs but not in the 3 cases with simple TEC. Frozen section examinations during TSS could be valuable for intraoperative assessment of the cyst [29, 30].

Since the first description of TEC by Dockerty and Priestly in 1942 [1] the histogenesis of the lesion remained poorly understood. Many clinical features, particularly age of predisposition, association with undescended testis and preponderance of the right side favour the origin from teratoma. Other findings (mainly the absence of GCNis and the very slow growth rate) argue against this theory. The WHO 2016 Classification System of Tumours defined TEC (and some other rare entities) as a specialized form of prepubertal-type teratoma that is unrelated to germ cell neoplasia in situ” [2] and that may occur in adulthood, too. Similarly, other prepubertal types of teratoma have been documented to sporadically occur in adult patients [31]. Thus, the simple TECs reported herein would represent teratomas of prepubertal type as described in 2016 WHO classification. The two cases with “complex TECs” reported herein would represent teratomas of postpubertal type (ICD-O 9080/3) according to the WHO classification with the “/3″ coding for malignancy. This type of epidermoid cysts thus represents a specialised differentiation of teratoma analogous to the development of other benign structures within teratomas, e.g. hairs or teeth. The two types of TECs can be clearly differentiated with regard to the histogenesis according to the WHO classification. However, from a clinical point of view, this differentiation is not really advantageous. Therefore, it is suggested to define two distinct classes of TEC, clinically, the first representing the more common “simple TEC” without malignant features (i.e. without accompanying GCNis) and the second encompassing the “complex TEC” cases with accompanying GCT.

## Conclusion

Testicular epidermoid cysts may occur in two different forms, the benign subtype, called “simple TEC”, clinically, and the other subtype representing an epidermoid cyst that occurs in association with invasive germ cell tumours (GCNIS), called the “complex TEC”, clinically. Urologic surgeons must be aware of the second type because conservative surgery would be inappropriate in these cases.
